# Developing an instrument to measure the quality of social work cancer counseling regarding return to work — psychometric properties of the German Quality of Cancer Counseling Questionnaire-Return to Work (QCCQ-W)

**DOI:** 10.1007/s00432-024-06040-6

**Published:** 2024-12-05

**Authors:** Clara Breidenbach, Sabine Schneider, Marie Rösler, Nicole Ernstmann, Paula Heidkamp, Lina Heier, Kati Hiltrop, Sophie Schellack, Johannes Soff, Johanna Weiss, Christoph Kowalski

**Affiliations:** 1 German Cancer Society, Berlin, Germany; 2 University of Cologne, Faculty of Medicine and University Hospital Cologne, Institute of Medical Sociology, Health Services Research and Rehabilitation Science, Chair of Health Services Research, Cologne, Germany; 3 University of Esslingen, Esslingen, Germany; 4 Working Group on Social Work in Oncology (ASO) in the Germany Cancer Society, Berlin, Germany; 5 Department of Psychosomatic Medicine and Psychotherapy, Center for Health Communication and Health Services Research, Bonn University Hospital, Bonn, Germany; 6 Department of Clinical Pharmacy and Toxicology, Maastricht University Medical Center, Maastricht, The Netherlands; 7 CARIM School for Cardiovascular Disease, Maastricht University, Maastricht, The Netherlands

**Keywords:** Cancer counseling, Instrument, Psychosocial, Social work

## Abstract

**Purpose:**

Counseling by social workers can be of great relevance supporting cancer survivors with their psychosocial challenges such as returning to work. However, an instrument for assessing the quality between social worker and client in the cancer counseling setting regarding return to work is not yet available. This study was carried out to develop and validate an instrument for this purpose.

**Methods:**

A questionnaire was developed in collaboration with cancer counseling experts. Data collection took place in 19 outpatient cancer counseling centers in Germany. Cancer survivors seeking advice regarding their occupational situation were asked to complete the questionnaire 3 months after starting counseling. Exploratory factor analysis (*n* = 229) and confirmatory factor analysis (*n* = 216) in two distinct samples, as well as validity and reliability tests, were performed.

**Results:**

Exploratory factor analysis suggested a two-component solution. Confirmatory factor analysis confirmed this solution, with a satisfactory model fit (CFI = 0.995, RMSEA = 0.049, SRMSR = 0.036). The components identified included six items with three items each and were termed “1. relationship building” and “2. competent support.” The components had good internal consistency (Cronbach’s α between 0.84 and 0.92) and test–retest reliability (1. *r*(30) = 0.49, *p* = 0.005, 2. *r*(30) = 0.89, *p* < 0.001). Significant correlations with other constructs measuring satisfaction with counseling and its usefulness indicated moderate to good construct validity (r between 0.36 and 0.77, *p* < 0.001).

**Conclusion:**

The questionnaire developed shows satisfactory psychometric properties. It is an evaluation tool specific for cancer counseling regarding return to, since it will initially be used to evaluate an intensified program for supporting cancer survivors returning to work after treatment. It may also be tested as an instrument for quality assurance and management in general cancer counseling in the future.

## Introduction

Cancer and other severe physical diseases may be accompanied by a serious psychosocial burden. It is not only distress, anxiety, and depression that present issues to cancer survivors (Mehnert et al. 2014, 2018); the illness also has a major impact on survivors’ participation in society, including their role and social functioning, financial difficulties, and last but not least issues revolving around (returning to) work and early retirement (Singer et al. 2014; Carrera et al. 2018; Arndt et al. 2019). Psychosocial counseling by social workers addresses these issues and can play an important role in supportive care for cancer patients.

However, evidence-based practice and outcome research have only recently started to develop in oncology social work in comparison with other oncological disciplines. Whereas some disciplines in cancer care have developed measures for quality evaluation, such as quality indicators, there is a lack of evaluation measures for social work counseling in oncology (Mahesh et al. 2024). According to Donabedian (1988), quality in health care is multi-dimensional including aspects on structure, process and outcome level. One aspect that has been linked to quality in helping professions such as social work is the relationship between the helper and the client (Horvath 2018). For example, in psychotherapy, meta-analyses have shown positive associations between the strength of the relationship and the outcome of therapy (Flückiger et al. 2018; Baier et al. 2020). The role of the relationship between the client and helper has also been receiving increasing attention in other professions such as physiotherapy and rehabilitation (Babatunde et al. 2017; Søndenå et al. 2020), as well as nursing (Hartley et al. 2020). In social work, the relationship between the client and the social worker is also considered essential (Schäfter 2010; Sinai-Glazer 2020), but empirical research is limited. Besides establishing a respectful and appreciative atmosphere, however, the provision of expert knowledge and competent advice by the social worker regarding the client’s specific situation is also perceived as crucial for the quality of the counseling (Schneider and Walther 2019). These components might reflect aspects of quality on different levels according to Donabedian.

Various instruments have been developed for measuring the quality of helping professions, particularly in general psychotherapy, including the Helping Alliance Questionnaire (Luborsky 2000) and the Working Alliance Inventory (Tracey and Kokotovic 1989). However, processes and outcomes of social work (in cancer counseling) cannot be equated with other helping professions such as general psychotherapy. This can be illustrated by items in existing instruments that are not fully applicable to the context of social work in cancer counseling: Items such as “As a result of the therapy I became clearer as to how I might be able to change” from the Working Alliance Inventory (Tracey and Kokotovic 1989), or “I feel now that I can understand myself and deal with myself on my own” from the Helping Alliance Questionnaire (Eich et al. 2018) demonstrate that these instruments are directed toward psychological and psychopathological challenges, whereas social work in cancer counseling has different focuses — for example, social security law — that are not covered by the above items. Therefore, this paper poses the following research question: What are the psychometric properties of a survey instrument developed for evaluating the quality of cancer counseling specific to return to work by a social worker? To the best of our knowledge, this will be the first instrument to explicitly measure this aspect.

## Methods

### Study design and data collection

The questionnaire was developed, and data collection for it took place, within the Cancer Rehabilitation Support by Cancer Counseling Centers (CARES) study. This is a feasibility study with a quasi-experimental pre/post design that aims to develop, implement, and evaluate a counseling intervention in outpatient cancer counseling centers (OCCs) that focuses on returning to work (Hiltrop et al. 2023). Data collection took place in 19 OCCs in Germany, with two study groups of cancer survivors seeking advice regarding their occupational situation. OCCs were selected from members of the Federal Working Group for Outpatient Cancer Counselling Centres (Bundesarbeitsgemeinschaft für Krebsberatungsstellen - BAK). BAK board members suggested member OCCs they considered prepared for timely participation in the study, then those recommended OCCs were selected according to geography (distribution over country, urbanity). Preselected OCCs were invited to participate in the project.

The first group of cancer survivors that participated in the study received regular counseling in the OCCs (comparison group, CG). Afterward, the second group of participants underwent the newly developed intervention (intervention group, IG). Participants in both groups were surveyed at the start of the counseling process after study enrolment (T0), 3 months after enrolment (T1), and up to 18 months (T1.2) after enrolment. Participants in the IG were also surveyed at the end of their counseling (T2), whereby T2 could either be before or after T1 because the end of the counseling was at an individual point of time. The study inclusion criteria were: having an oncological diagnosis, problems and/or counseling needs regarding the occupational situation, age over 18, sufficient German language skills, no cognitive limitations impeding participation in surveys or interviews, and informed consent. Eligibility criteria were checked by staff in the OCCs, eligible cancer survivors were then invited to participate in the study by the OCCs. T0 and T2 were handed over by the OCCs, T1 and T1.2 were sent postally to the participants by the study team. For T1 and T1.2 non-responders were reminded twice. Participants who did not respond for T1 also received an invitation to fill in T1.2. The study was approved by the Ethics Committee of the Medical Faculty of the University of Bonn (061 − 22; April 9, 2022).

### Questionnaire development

The questionnaire development followed Donabedian’s approach (Donabedian 1988) that quality might include aspects on different levels. In order to cover different processes and outcomes, we designed the questionnaire based on central components of social work in cancer counseling. According to the Working Group on Social Work in Oncology in the German Cancer Society (ASO) expert panel, these central components are: 1, establishing an open communication situation and relationship building; 2, clarification and negotiation processes; 3, provision of information; 4, support and accompaniment in the process; and 5, evaluation and conclusion of counseling. The initial main categories in the German Quality of Cancer Counseling Questionnaire-Return to Work (QCCQ-W) were therefore created based on these five central measures of cancer counseling, and in consultation with experts on and practitioners of cancer counseling of the ASO (see Table 2). In a meeting, two persons from the research team and two members from the ASO discussed concepts and hypotheses as well as formulated an initial pool of 21 items in the individual categories considering existing alliance instruments (Tracey and Kokotovic 1989; Eich et al. 2018) and the five central measures of counseling. This procedure has been described as rational method in questionnaire design elsewhere (Oosterveld et al. 2019).

The items were then pretested in a cognitive think-aloud interview with a cancer survivor for testing comprehensibility and content relevance, which resulted in slight adaptations of the wording because no need for other adaptations e.g. exclusion of items or inclusion of further topics was identified. The questionnaire that was entered into the survey contained 21 items. The analysis presented here only includes 20 items, since the last item had too many missing responses (59%, “If your consultation has already been completed (if not, please proceed to the next question): My counselor and I have discussed whether my goals were achieved”). An explanation for the high number of missing values for the last item might be that most of the participants had not yet completed the counseling process when responding to the survey and therefore skipped the item. Items in the QCCQ-W were measured on a scale from 1 (= “does not apply at all” [“trifft überhaupt nicht zu”]) to 7 (= “fully applies” [“trifft voll und ganz zu”]). The QCCQ-W was measured at T1. The QCCQ-W was developed and analyzed in German.

The characteristics of the participants, listed in Table 1, were documented at T0.

**Table 1:** Characteristics of the participants

	Comparison group (*n* = 229)	Intervention group (*n* = 216)
Age (years): mean, median (IQR)	51, 53 (46, 59)	50, 52 (44, 58)
Data missing	0	1
Gender, *n* (%)		
Female	175 (76.4)	181 (83.8)
Male	53 (23.1)	34 (15.8)
Diverse	1 (0.4)	0
Data missing	0	1
Highest educational qualification, *n* (%)		
No school-leaving qualification (yet)	1 (0.4)	2 (0.9)
Lower secondary school	17 (7.4)	17 (7.9)
Intermediate secondary school	87 (38.0)	74 (34.2)
Entrance certificate for university of applied sciences	17 (7.4)	2 (12.5)
Entrance certificate for university	105 (45.9)	93 (43.1)
Other	0	2 (0.9)
Data missing	0	1 (0.4)
Months since first cancer diagnosis: *n*; mean, median (IQR)	229; 23, 11 (5, 24)	219; 30, 12 (7, 23)
Tumor type, *n* (%, multiple answers possible)		
Breast (C50)	106 (46.3)	105 (48.6)
Colorectum/anus (C17–21)	15 (6.6)	15 (6.9)
Uterus, cervix, endometrium (C53–55)	10 (4.4)	11 (5.1)
Prostate (C61)	10 (4.4)	6 (2.8)
Leukemia (C91–96)	6 (2.6)	10 (4.6)
Skin/melanoma (C43, C44)	8 (3.5)	7 (3.2)
Other	160 (53.5)	60 (27.8)
Data missing	2 (0.1)	8 (3.7)

IQR, interquartile range

### Statistical analysis

Since our goal was to test the reliability and validity of the QCCQ-W based on its items, we followed classical test theory approach (Cappelleri et al. 2014). Furthermore, we expected the QCCQ-W to be multidimensional (different components of social work counseling) and reflective (items are manifestations of the different components). Therefore, according to COSMIN checklist (Mokkink et al. 2019), we decided to perform factor analyses. Exploratory factor analysis (EFA) was carried out to explore the data structure using data from the comparison group (CG) at T1. To identify whether there were sufficiently large correlations between the items for an EFA, Bartlett’s test of sphericity was run. The suitability for EFA of each item and the set of items was then evaluated using the measure of sampling adequacy (MSA; > 0.5) and the Kaiser–Meyer–Olkin measure (KMO; > 0.5 mediocre, > 0.7 good, > 0.8 great, > 0.9 superb; Field et al. 2012). Afterward, a principal component analysis (PCA) was conducted with orthogonal rotation (Varimax). The final number of extracted factors was led by the Kaiser criterion (eigenvalues > 1), inspection of the scree plot, as well as theoretical considerations (Field et al. 2012). Factor loadings > 0.4 and cross-loadings < 0.4 were considered acceptable (Field et al. 2012; Hair et al. 2014).

In order to validate the structure identified in the EFA, a confirmatory factor analysis (CFA) was conducted using data from the intervention group (IG) at T1. Model fit was evaluated by a combination of the following criteria: comparative fit index (CFI, ≥ 0.95), root mean square error of approximation (RMSEA, < 0.08), and standardized root mean square residual (SRMSR, < 0.08) (Hu and Bentler 1999; Hair et al. 2014).

To assess the construct validity of the QCCQ-W, bivariate correlations (Pearson) with other constructs measuring satisfaction with and usefulness of the counseling were performed. A significant positive correlation was expected to indicate adequate validity (Hobart et al. 2004; Mokkink et al. 2010). The instrument’s reliability in terms of internal consistency was examined using Cronbach’s α (> 0.8 good; > 0.9 may suggest redundancies) (Tavakol and Dennick 2011; Field et al. 2012). Test–retest reliability was assessed by correlating (Pearson) the QCCQ-W scores from the IG at T1 and T2.

Missing data within the items in the QCCQ-W were deleted listwise, leading to *n* = 229 cases in the CG and *n* = 216 cases in the IG. All data analyses were performed using RStudio 4.2.2 with the packages nFactors (Raiche and Magis 2022) and psych (Revelle 2023) for EFA, as well as lavaan for CFA (Rosseel 2012).

## Results

### Sample characteristics

A total of 229 participants in the CG and 216 participants in the IG completed the questionnaire at T1. The characteristics of the participants are presented in Table 1. The respondents in the CG had a mean age of 50 years and those in the IG had a mean age of 51 years. Both groups included more women (CG 76.4%, IG 83.8%), and the highest educational qualification for most respondents was a university entrance certificate (CG 45.9%, IG 43.1%). The majority of the participants in both groups had been diagnosed with breast cancer (CG 46.3%, IG 48.6%). The period between the first cancer diagnosis and enrolment in the study was slightly shorter in the CG, with a mean of 23 months, in comparison with the IG at 30 months. T2 was completed by 42 participants in the IG.

### Exploratory factor analysis

The Kaiser–Meyer–Olkin measure verified the sampling adequacy for the PCA, at KMO = 0.94, and all MSA values for individual items were > 0.90. Bartlett’s test of sphericity was significant, χ² (190) = 4046.626, *p* < 0.0001. An initial analysis was run to obtain eigenvalues for each component in the data. Two components had eigenvalues over Kaiser’s rule of 1 and in combination explained 65.9% of the variance. The scree plot showed an inflexion point that also justified retaining two components. With regard to the factor loadings as well as considerations in respect of whether the items fit to each other contentwise (not too similar, cover all relevant aspects), two components with four items each were retained in the final analysis. The retained items (factor 1– “relationship building”: 3, 4, 5, 11; factor 2– “competent support”: 12, 13, 16, 20, see Table 2 for details on items) had loadings ranging from 0.73 to 0.88.

**Table 2:** Descriptive results from the exploratory factor analysis (EFA)

Item	M (SD)	Factor 1 loading	Factor 2 loading
Establishment of an open communication situation and relationship building			
1 It was easy for me to talk about my own concerns during the counseling session. [In der Beratung fiel es mir leicht, zu erzählen, was mich beschäftigt.]	6.3 (1.1)	0.52	0.14
2 My concerns were correctly understood. [Meine Anliegen wurden richtig erfasst.]	6.5 (0.9)	0.83	0.28
**3 The problems bothering me were taken seriously. [Meine Belastungen wurden ernst genommen.]**	**6.7 (0.7)**	**0.78**	**0.19**
**4 My counselor had high regard for what I’ve achieved since the cancer diagnosis. [Meine Beraterin/ mein Berater hat wertgeschätzt**,** was ich bisher seit der Krebserkrankung geschafft habe.]**	**6.5 (0.9)**	**0.70**	**0.25**
**5 I was able to trust my counselor. [Ich konnte meiner Beraterin/ meinem Berater Vertrauen entgegenbringen.]**	**6.6 (0.8)**	**0.88**	**0.24**
6 I thought my counselor was competent. [Ich fand meine Beraterin/ meinen Berater kompetent.]	6.5 (0.9)	0.82	0.35
7 My counselor gave me support for tackling things myself. [Meine Beraterin/ mein Berater hat mich unterstützt, selbst Dinge anzugehen.]	6.2 (1.1)	0.69	0.43
8 The counseling helped me make my own decisions. [Die Beratung hat mir geholfen, eigene Entscheidungen zu treffen.]	6.1 (1.2)	0.66	0.51
9 My counselor made plenty of time for me. [Meine Beraterin/ mein Berater hat sich Zeit für mich genommen.]	6.6 (0.9)	0.68	0.29
10 I had the feeling I could contact my counselor at any time. [Ich hatte das Gefühl, dass ich mich jederzeit bei meiner Beraterin/ meinem Berater melden konnte.]	6.4 (1.2)	0.60	0.29
**11 I felt there was a good atmosphere in the counseling sessions. [Die Atmosphäre in den Gesprächen habe ich als angenehm erlebt.]**	**6.6 (0.7)**	**0.85**	**0.22**
Clarification and negotiation processes			
**12 The counseling helped me clarify my future working career. [Die Beratung hat mir geholfen**,** meine berufliche Zukunft zu klären.]**	**5.2 (1.8)**	**0.24**	**0.84**
**13 The counseling helped me formulate goals. [In der Beratung ist es mir gelungen**,** Ziele zu formulieren.]**	**5.6 (1.6)**	**0.25**	**0.84**
14 The counseling helped me identify ways of achieving the goals. [In der Beratung ist es mir gelungen, Möglichkeiten zu finden, diese Ziele zu erreichen.]	5.4 (1.6)	0.26	0.86
15 My own abilities and limitations were taken into account during the counseling. [In der Beratung wurden meine eigenen Möglichkeiten und Grenzen berücksichtigt.]	6.0 (1.3)	0.47	0.67
**16 The counseling helped me coordinate my health situation and my work situation better. [Die Beratung hat mir geholfen**,** meine gesundheitliche und berufliche Situation besser miteinander zu vereinbaren.]**	**5.3 (1.7)**	**0.27**	**0.85**
Provision of information			
17 The information I received from my counselor was appropriate to my own situation. [Ich habe Informationen von meiner Beraterin/ meinem Berater erhalten, die zu meiner Situation gepasst haben.]	6.1 (1.3)	0.60	0.54
18 The information I received from my counselor was new for me. [Ich habe neue Informationen von meiner Beraterin/meinem Berater erhalten.]	5.9 (1.5)	0.44	0.52
Support and accompaniment in the process			
19 My counselor gave me committed support. [Meine Beraterin/ mein Berater hat mich engagiert unterstützt.]	6.2 (1.2)	0.63	0.55
**20 My counselor and I regularly discussed whether my goals were still up to date. [Meine Beraterin/ mein Berater und ich haben regelmäßig besprochen**,** ob meine Ziele noch aktuell sind.]**	**4.6 (2.2)**	**0.17**	**0.73**
Evaluation and conclusion of counseling			
21 If the counseling is already completed: My counselor and I have discussed whether my goals were achieved. [Wenn Ihre Beratung bereits abgeschlossen ist: Meine Beraterin/ mein Berater und ich haben besprochen, ob meine Ziele erreicht wurden.]	Excluded from analysis		

Notes *n* = 229 (comparison group). Response categories ranged from 1 (= “does not apply at all” [“trifft überhaupt nicht zu”]) to 7 (= “fully applies” [“trifft voll und ganz zu”]). M, mean, SD, standard deviation; retained items are highlighted in bold

The introductory text for the items read: “We present here a few statements about the counseling provided by the cancer counseling center. Please indicate the extent to which you agree with the statements. “7” indicates that a statement fully applies, “1” indicates that a statement does not apply at all. You can grade your assessment using the boxes in between. Please place one cross for each statement. Some of the statements refer to the counselor. If you received counseling from several people, please think about the person who advised you the most.” [Wir stellen Ihnen nun einige Aussagen zur Beratung durch die Krebsberatungsstelle vor. Bitte geben Sie an, inwieweit Sie den Aussagen zustimmen. „7“ bedeutet, dass eine Aussage voll und ganz zutrifft, „1“ bedeutet, dass eine Aussage überhaupt nicht zutrifft. Mit den Kästchen dazwischen können Sie Ihre Einschätzung abstufen. Bitte setzen Sie pro Aussage ein Kreuz. Bei einigen Aussagen ist von Ihrer Beraterin/ Ihrem Berater die Rede. Wenn Sie durch mehrere Personen beraten wurden, denken Sie bitte an die Person, von der Sie am meisten beraten wurden]

### Confirmatory factor analysis

**Fig. 1:**
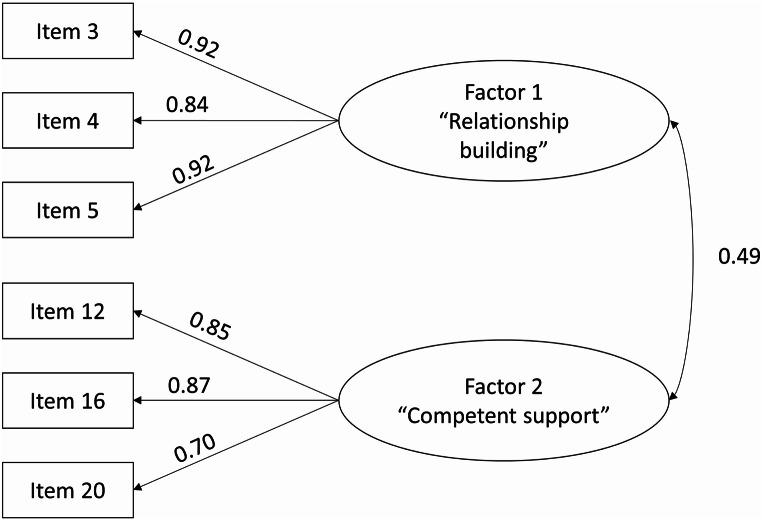
Results from the confirmatory factor analysis (modified model)

The identified two-factor structure of the PCA with data from the CG was replicated with a CFA with data from the IG (original model). After removal of items 11 (“I felt there was a good atmosphere in the counseling sessions”) and 13 (“The counseling helped me formulate goals”) due to insufficient support from the CFA for these items (modified model, Fig. 1), indices supported the global model fit: CFI = 0.995, RMSEA = 0.049, SRMSR = 0.036 (Table 3). Local model fits did not indicate any potential improvements for the modified model. The modified model contained the following items: relationship building– 3. The problems bothering me were taken seriously.; 4. My counselor had high regard for what I’ve achieved since the cancer diagnosis.; 5. I was able to trust my counselor.; competent support– 12. The counseling helped me clarify my future working career.; 16. The counseling helped me coordinate my health situation and my work situation better.; 20. My counselor and I regularly discussed whether my goals were still up to date.

**Table 3:** Model fit indicators in the confirmatory factor analysis (CFA; *n* = 226)

Thresholds	CFI ≥ 0.95	RMSEA < 0.06	SRMSR < 0.08
Original model	0.989	0.109	0.068
Modified model	0.995	0.049	0.036

CFI, comparative fit index; RMSEA, root mean square error of approximation; SRMSR, standardized root mean square residual

### Construct validity and reliability

The factor “relationship building” showed a significant positive correlation with satisfaction with counseling, at *r*(226) = 0.55, *p* < 0.001 in the CG and *r*(212) = 0.36, *p* < 0.001 in the IG, and with usefulness of the counseling, at *r*(226) = 0.39, *p* < 0.001 in the CG and *r*(212) = 0.42, *p* < 0.001 in the IG. The factor “competent support” also showed a significant positive correlation with the satisfaction with counseling measure, at *r*(226) = 0.47, *p* < 0.001 in the CG and *r*(212) = 0.47, *p* < 0.001 in the IG, and with usefulness of the counseling, at *r*(226) = 0.70, *p* < 0.001 in the CG and *r*(212) = 0.77, *p* < 0.001 in the IG. Internal consistency was good: The factor “relationship building” showed a Cronbach’s α of 0.85 in the first sample (CG) and 0.92 in the second sample (IG). The factor “competent support” showed a Cronbach’s α of 0.86 in the first sample (CG) and 0.84 in the second sample (IG). “Relationship building”, at *r*(30) = 0.49, *p* = 0.005, and “competent support”, at *r*(30) = 0.89, *p* < 0.001, both correlated positively between measurements at T1 and T2 in the IG.

## Discussion

The aim of this study was to develop an instrument for measuring the quality between social worker and client in cancer counseling.

Following the exploratory factor analysis, the QCCQ-W’s initial structure of 21 items and five factors was reduced to eight items and two factors (original model). The theoretically postulated five-factor structure was therefore not confirmed psychometrically. The first factor, “relationship building,” represents the concept of establishing an open communication environment during cancer counseling: accepting the client’s perspective, with an open outcome and with the counseling being guided by a client-based approach. The items included under this factor measure whether clients feel they are being taken seriously and valued by the social worker, as well as whether the client perceives the counseling atmosphere as pleasant. The second factor is termed “competent support,” and the items included under it revolve around whether the situation for the client has been clarified and improved through the counseling, as well as whether the social worker has constantly reevaluated the client’s situation. This factor includes aspects of clarification and negotiation processes, as well as support and accompaniment processes, which — according to the expert social workers who were consulted — are important parts of the counseling process and require competence and specific know-how on the part of the social worker — regarding social security law, for example, which is particularly difficult for clients to comprehend and act on. These two factors together thus represent aspects of process as well as outcome quality, which are both relevant for quality of social work cancer counseling.

Since this is the first questionnaire measuring quality in the context of cancer counseling regarding return to work, the results of the present analysis can only be compared with findings from the psychotherapeutic context. A psychometric analysis of the German version of the Helping Alliance Questionnaire also revealed a possible two-factor structure, with one factor referring to the relation between therapist and client and the other factor referring to satisfaction with the outcome (Eich et al. 2018). A systematic review of the Working Alliance Inventory’s measurement properties shows a tendency toward a three-factor structure in the literature (Paap et al. 2022). This shows that the QCCQ-W does not strongly diverge from established alliance questionnaires at the psychometric level. At the content level, however, it differs from existing instruments that focus on psychopathological concerns by concentrating more on social work issues — e.g., “The counseling helped me coordinate my health situation and my work situation better.” Quality in social work cancer counseling has not been addressed in terms of validated questionnaires to our knowledge, however, it has been addressed in other quality endeavors: For example, a Project to Assure Quality Cancer Care (APAQCC) by the Association for Oncology Social Work (AOSW) aimed to examine the capacity of cancer programs to provide quality psychosocial support services, and to evaluate the implementation of distress screening (Zebrack et al. 2018). Within this project, an instrument (Oncology Social Work Intervention Index, OSWii) was developed especially for research to examine the impact of psychosocial interventions on patient outcomes that captures whether and how social work intervention addresses the psychosocial needs of patients and has the potential to demonstrate the value of oncology social work practice (Oktay et al. 2021). The index is filled out by social workers or researchers, thus, is not fully comparable to the QCCQ-W but both instruments reflect upon certain processes and practices of social work interventions.

The confirmatory factor analysis (CFA) confirmed the two-factor structure of the EFA but suggested three items per factor instead of four (modified model). The modified model showed an adequate fit with the data. Construct validity was indicated, since the QCCQ-W correlated significantly with satisfaction with and usefulness of the counseling, however correlations between the tested constructs were only moderate to good (Evans 1996). The instrument’s reliability in terms of internal consistency was very good for both factors in both samples, also confirmed by the test–retest reliability results.

With regard to limitations of the present study, it should be noted that the sample includes above-average numbers of women and of individuals with higher educational levels in comparison with cancer patients in general. However, this represents the general client base for cancer counseling centers in Germany (Ernst et al. 2016). In addition, only one cognitive think-aloud interview took place before data collection which means that we had only received one feedback comprehensibility and relevance. More cognitive think-aloud interviews might have resulted in other formulations or results on comprehensibility and relevance. The QCCQ-W was also developed in the special setting of the CARES study, that is to say survivors especially seeking advice regarding in the context of professional reintegration, which involved longer-term counseling than is generally offered in outpatient cancer counseling. The QCCQ-W’s validity beyond the CARES setting should therefore be investigated outside of context of work-related counselling. Also, two items in factor 2 ask specifically whether the counseling was successful in terms of sorting out work-related issues. In order to provide an instrument that can be widely used for general topics of cancer counseling, items and samples should be generalized. We have already developed generalized items that might be implemented in the QCCQ-W in a follow-up survey or other projects. In the future, the clients’ perspective measured with the QCCQ-W could additionally be compared with the social workers’ perspective. The choice of using a Likert-scale can further be discussed, since Likert-scales are known to generate an acquiescence-bias. We have tried to counteract this bias by using a 7-point-scale which has been described as best practice in literature (Franzen 2019) allowing enough variance as well as a middle category leaving respondents the possibility of not choosing between disapproval and approval.

The QCCQ-W instrument was developed as a quality assurance instrument for assessing social worker counseling regarding return to work in OCCs. The final instrument developed consists of six items within two factors termed “relationship building” and “competent support.” It has adequate and reliable psychometric properties. The QCCQ-W represents a good starting point for developing quality assurance tools for outpatient cancer counseling care– It is especially designed for counseling in work-related issues and can be further developed for general cancer counseling by adapting the two work-related items.

## Data Availability

Data is not available.
